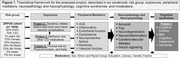# Design, Characteristics, and Resources from the Diabetes Prevention Program Outcomes Study (DPPOS) AD/ADRD project

**DOI:** 10.1002/alz.086325

**Published:** 2025-01-09

**Authors:** Jose A. Luchsinger, Diabetes Prevention Program Research Group

**Affiliations:** ^1^ Columbia University Medical Center, New York, NY USA

## Abstract

**Background:**

Persons with prediabetes and type 2 diabetes (T2D) are known to have higher risk of cognitive impairment (CI), including age‐related cognitive decline, mild cognitive impairment (MCI), and dementia; however, the characteristics of CI and the determinants and mechanisms in prediabetes and T2D remain unclear. Addressing these gaps is critical since over half of the U.S. population aged 60 years and older, who are at risk for AD/ADRD, have prediabetes or T2D.

**Methods:**

The Diabetes Prevention Program (DPP) was a randomized clinical trial among adults with prediabetes which started in 1996 and demonstrated T2D risk reduction with intensive lifestyle or metformin compared with placebo. The DPP Outcomes Study (DPPOS) is a 25‐center observational follow‐up study. Combined, DPP/DPPOS has followed the original cohort for a mean of 23 (range 21‐25) years with extensive longitudinal phenotyping and a comprehensive data and biospecimen repository. To study AD/ADRD in DPPOS we have implemented (a) the NACC UDS version 3 for the adjudication of MCI and dementia; (b) measurement of plasma biomarkers of amyloid, neurodegeneration, and neuroinflammation; (c) examination of plasma measures of brain insulin signaling via neuron‐derived exosomes; and (d) brain MRI and amyloid PET scanning. Figure 1 describes the constructs of the four interrelated projects of DPPOS‐AD/ADRD. Data and biospecimens will be deposited to relevant repositories and available to researchers.

**Results:**

The diverse cohort, originally selected to have prediabetes and at high risk of developing T2D, currently includes 1654 participants, 70% of whom have developed T2D of known duration. Forty‐eight percent are non‐White, and 73% are women. The mean age of the cohort in 1996 was 51 years and now 73 years, with 84% ≥65y. We incorporated the standardized NACC UDS protocol for ascertainment of cognitive status with full fidelity beginning in November 2022, including recorded neurologic exams. Brain imaging began in summer 2023, and exosome extraction began in early 2024.

**Conclusions:**

State of the art procedures, typically used in AD/ADRD research, have been implemented in a large multi‐center non‐AD/ADRD cohort. The proposed measures will supplement those already gathered and provide novel insight into CI in people with prediabetes and T2D.